# Diagnosis and treatment of musculoskeletal chest pain: design of a multi-purpose trial

**DOI:** 10.1186/1471-2474-9-40

**Published:** 2008-03-31

**Authors:** Mette J Stochkendahl, Henrik W Christensen, Werner Vach, Poul Flemming Høilund-Carlsen, Torben Haghfelt, Jan Hartvigsen

**Affiliations:** 1 Nordic Institute of Chiropractic and Clinical Biomechanics, Part of Clinical Locomotion Science, Odense, Denmark; 2 Institute of Sports Science and Clinical Biomechanics, University of Southern Denmark, Odense, Denmark; 3 Department of Statistics, University of Southern Denmark, Odense, Denmark; 4 Department of Nuclear Medicine, Odense University Hospital, Denmark, Odense, Denmark; 5 Department of Cardiology, Odense University Hospital, Denmark, Odense, Denmark

## Abstract

**Background:**

Acute chest pain is a major health problem all over the western world. Active approaches are directed towards diagnosis and treatment of potentially life threatening conditions, especially acute coronary syndrome/ischemic heart disease. However, according to the literature, chest pain may also be due to a variety of extra-cardiac disorders including dysfunction of muscles and joints of the chest wall or the cervical and thoracic part of the spine. The diagnostic approaches and treatment options for this group of patients are scarce and formal clinical studies addressing the effect of various treatments are lacking.

**Methods/Design:**

We present an ongoing trial on the potential usefulness of chiropractic diagnosis and treatment in patients dismissed from an acute chest pain clinic without a diagnosis of acute coronary syndrome. The aims are to determine the proportion of patients in whom chest pain may be of musculoskeletal rather than cardiac origin and to investigate the decision process of a chiropractor in diagnosing these patients; further, to examine whether chiropractic treatment can reduce pain and improve physical function when compared to advice directed towards promoting self-management, and, finally, to estimate the cost-effectiveness of these procedures. This study will include 300 patients discharged from a university hospital acute chest pain clinic without a diagnosis of acute coronary syndrome or any other obvious cardiac or non-cardiac disease. After completion of the clinic's standard cardiovascular diagnostic procedures, trial patients will be examined according to a standardized protocol including a) a self-report questionnaire; b) a semi-structured interview; c) a general health examination; and d) a specific manual examination of the muscles and joints of the neck, thoracic spine, and thorax in order to determine whether the pain is likely to be of musculoskeletal origin. To describe the patients status with regards to ischemic heart disease, and to compare and indirectly validate the musculoskeletal diagnosis, myocardial perfusion scintigraphy is performed in all patients 2–4 weeks following discharge. Descriptive statistics including parametric and non-parametric methods will be applied in order to compare patients with and without musculoskeletal chest pain in relation to their scintigraphic findings. The decision making process of the chiropractor will be elucidated and reconstructed using the CART method. Out of the 300 patients 120 intended patients with suspected musculoskeletal chest pain will be randomized into one of two groups: a) a course of chiropractic treatment (therapy group) of up to ten treatment sessions focusing on high velocity, low amplitude manipulation of the cervical and thoracic spine, mobilisation, and soft tissue techniques. b) Advice promoting self-management and individual instructions focusing on posture and muscle stretch (advice group). Outcome measures are pain, physical function, overall health, self-perceived treatment effect, and cost-effectiveness.

**Discussion:**

This study may potentially demonstrate that a chiropractor is able to identify a subset of patients suffering from chest pain predominantly of musculoskeletal origin among patients discharged from an acute chest pain clinic with no apparent cardiac condition. Furthermore knowledge about the benefits of manual treatment of patients with musculoskeletal chest pain will inform clinical decision and policy development in relation to clinical practice.

**Trial registration:**

NCT00462241 and NCT00373828

## Background

Acute chest pain is believed to be one of the most common reasons for hospital admission in Denmark [1,2]. Figures from the United States show that chest pain is the second most common reason for emergency department visits, accounting for 5.4% or more than 4 million visits per year [3]. The primary concern in these cases is of cause cardiac disease, but in about 50% of cases the aetiology appear to be non-cardiac [4,5], and in around 20% of the patients admitted to chest pain clinics no definitive diagnosis can be made [6].

Chest pain patients with normal coronary perfusion have an excellent prognosis for survival, and a future risk of cardiac morbidity similar to that reported in the background population [7-9]. However, about three quarters of patients with undiagnosed chest pain continue to suffer from residual pain with large personal and socio-economic consequences in terms of anxiety, fear of undiagnosed heart disease, loss of daily function and working capacity, and re-admissions to the hospital [10-16]. Chest pain differential diagnoses include primarily pulmonary, gastrointestinal, psychosocial, or musculoskeletal problems. Musculoskeletal problems alone accounts for 5–20% of the total number of admissions in acute chest pain clinics [16-18]. Hence, the musculoskeletal system is a recognized possible source of pain in patients with chest pain, even if no standardized criteria for the diagnosis exist at this point.

An extensive body of literature addresses patient assessment and management protocols for patients presenting with chest pain, but these focus primarily on cardiopulmonary [19-22], gastro-oesophageal [5,23], and psychological conditions [11,24,25], and protocols aiming at diagnosis of musculoskeletal chest pain remain scarce, and the effect of treatment strategies, including medical treatment (oral anti-inflammatory drugs), exercise (strength and/or stretching), advice, and manual approaches have not been evaluated. To our knowledge, only one non-randomized study deals with manual examination and treatment of patients with musculoskeletal chest pain [26,27]. In this study, an examination program consisting of a general health examination and a specific manual examination of the thorax and cervico-thoracic part of the spine was developed for a population of patients with suspected or known stable angina pectoris referred to a tertiary hospital for coronary angiography [26]. The examination program together with the detailed case history was applied by a chiropractor to make a diagnosis of discomfort from the musculoskeletal system, cervico-thoracic angina (CTA). In the absence of a true golden standard to validate the CTA diagnosis, myocardial perfusion scintigraphy (MPS) was used as a *by proxy *measure of validity with some success: Eighty percent of the CTA-positive patients had normal perfusion compared to 50% in the CTA-negative group. Moreover, results indicated that patients with CTA may benefit from chiropractic treatment.

We therefore decided to perform a multi-purpose clinical trial consisting of 1) a prospective, population-based, diagnostic evaluation study, 2) a single-blinded, randomized clinical trial (RCT), and 3) a cost-effectiveness analysis alongside the RCT.

The aims are:

• To determine the proportion of patients discharged from a university hospital chest pain clinic in whom their chest pain may be of musculoskeletal rather than cardiac origin. Specifically, we wish to determine the prevalence and character of musculoskeletal chest pain, and to describe cardiac status with respect to ischemic heart disease.

• To investigate the diagnostic decision making process of a chiropractor in these patients, using MPS as an indirect measure of validity.

• To determine the relative clinical effectiveness of chiropractic manual treatment versus advice directed towards promoting self-management using pain and patient-rated outcomes as primary outcome measures. Finally, we will estimate the cost-effectiveness of these procedures.

## Methods/Design

This clinical trial is being conducted at Odense University Hospital in Odense, Denmark. The study began in 2006, and is ongoing. Approval has been granted by the regional ethics committee for Funen and Vejle Counties, Denmark, approval number #VF 20060002, and informed consent is obtained from all participants.

### Study population

Three hundred consecutive patients with an episode of suspected non-cardiac acute chest pain are being recruited among patients discharged from an acute chest pain clinic situated at a large specialized cardiology department. All patients have undergone a standardized evaluation program at the chest pain clinic ruling out any obvious and significant cardiac or non-cardiac disease, including acute coronary syndrome. Following discharge from the chest pain clinic, all patient records are screened for the inclusion and exclusion criteria into the present study, and potential participants are contacted personally or by telephone and invited to participate.

### Inclusion/exclusion criteria

The inclusion and exclusion criteria are presented in Table 1.

**Table 1:** Inclusion and exclusion criteria.

**Inclusion criteria**	**Exclusion criteria**
*To be included in the project the participant must*	*Patients will not be included if any of the following conditions are present*
- Have chest pain as their primary complaint.	- Acute coronary syndrome.
- Have an acute episode of pain of less than 7 days duration before admission.	- Previous Percutaneous Coronary Intervention or Coronary Artery By-pass Grafting.
- Consent to the standardized evaluation program at the chest pain clinic.	- Chest pain from other definite cause, cardiac or non-cardiac. The condition must be verified clinically during admission (i.e. pulmonary embolism, pneumonia, dissection of the aorta, ...).
- Have pain in the thorax and/or neck.	- Inflammatory joint disease.
- Be able to read and understand Danish.	- Insulin dependent diabetes
- Be between 18 and 75 year of age.	- Fibromyalgia.
- Be a resident of the Funen County	- Malignant disease.
	- Apoplexy, dementia, or unable to cooperate.
	- Major osseous anomaly.
	- Osteoporosis.
	- Pregnancy.
	- Does not want to participate.
	- Other – the reason for non-inclusion will be registered.

A record will be kept of the number of subjects excluded from the study, as well as those who are eligible for inclusion and choose not to participate.

### Baseline measurements

Interested individuals are assessed at baseline within seven days of admission. First, they complete a questionnaire including information on pain, general health, occupation, education, physical and lifestyle factors, expectation to treatment outcome, and baseline values for the outcome measures.

Next, participants are examined using a standardized and previously validated study protocol [26]. The examination protocol consists of three parts:

*1) A semi-structured interview *including pain characteristics (frequency, duration, localization, provoking and relieving factors), symptoms from the lungs and gastrointestinal system, past medical history, height and weight, and risk factors for ischemic heart disease. Further, patients are classified into three types of chest pain: typical angina, atypical angina, or non-cardiac chest pain in accordance with Danish and international guidelines [19,21]. The patients are also classified into one of four classes of severity according to the criteria given by the Canadian Cardiovascular Society (CCS) [21,28]. Cardio-vascular performance is graded according to New York Heart Association (NYHA) [29].

*2) A general health examination *including blood pressure and pulse, heart and lung stethoscopy, abdominal palpation, neck auscultation, clinical signs of left ventricular failure, neurological examination of the upper and lower extremities in terms of reflexes, sensibility to touch, muscle strength, and an orthopaedic examination of the neck and shoulder joints in order to rule out nerve root compression syndromes.

*3) A specific manual examination *of the muscles and joints of the neck, thoracic spine and thorax, including active range of motion, manual palpation for muscular tenderness on 14 points of the anterior chest wall, palpation for segmental paraspinal muscular tenderness, motion palpation for joint-play restriction of the thoracic spine (Th1–8), and end play restriction of the cervical and thoracic spine [26].

The examination program together with the detailed case history will be applied by a chiropractor to make a diagnosis of pain from the musculoskeletal system, CTA, according to the previously established criteria [26].

**Figure 1:**
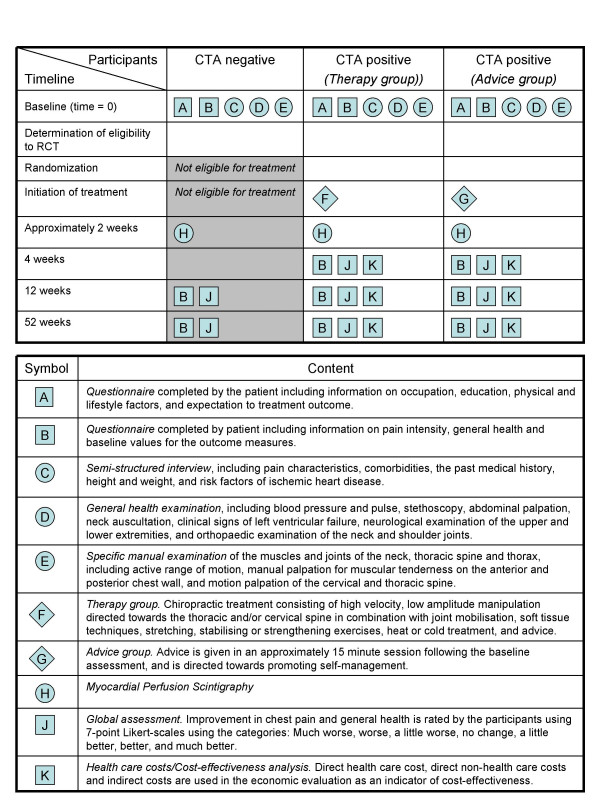
Evaluation, intervention and follow up. (Adapted from Perera et al. 2007).

The timeline and overview of data collection is shown in Figure 1 (adapted from Perera et al. (2007)[30]). The timeline is shown vertically, and allocation of participants to study groups horizontally.

### MPS

In order to evaluate the population in terms of ischemic heart disease all patients undergo MPS within two to four weeks following baseline evaluation. Using radionuclides the myocardial perfusion is evaluated to determine the presence of regional areas with decreased blood flow because of coronary artery disease. Detailed procedures for MPS are described in Appendix 1. MPS will also be used to compare and indirectly validate the musculoskeletal diagnosis.

### RCT

All CTA positive patients (estimated 120 out of the initial 300 patients) will be included in the RCT. The aim of this part of the study is to establish the effectiveness of chiropractic treatment including spinal manipulation versus advice to promote self-management. Participants are only eligible for inclusion in the RCT if they are CTA positive and the examining clinician decides that manipulation might be the appropriate treatment. Patients for whom manipulation is thought not to be indicated will not be included in the RCT.

### Randomization

The randomization sequence with a 1:1 allocation ratio has been computer generated by a researcher not involved in the project. Consecutively numbered sealed opaque envelopes containing the treatment allocation for each patient has been produced and eligible participants draw an envelope. The envelopes are arranged in blocks with varying block sizes. The examining clinician manages the hand over of the envelope to the participant, but is masked to the treatment allocation when determining eligibility to randomization.

### Treatments

Participants will be randomized to receive one of two treatments: A course of chiropractic treatment including spinal manipulation (therapy group) or advice promoting self-management (advice group).

#### Therapy group

Participants in the therapy group will be assigned to a chiropractor in their local community. Participating chiropractors will have a university chiropractic degree and at least five years of clinical experience. Each chiropractor chooses an individual treatment strategy based on a combination of their findings, the patient history, and pragmatic, routine practice. The treatment will be modified to accommodate the age and physical condition of each patient. The treatment must, however, include high velocity, low amplitude manipulation directed towards the thoracic and/or cervical spine in combination with any of the following: Joint mobilisation, soft tissue techniques, stretching, stabilising or strengthening exercises, heat or cold treatment, and advice. The protocol specifies up to ten treatment sessions of approximately 20 minutes, 1–3 times per week for four weeks, or until the patient is pain free if this occurs within less than four weeks. The type of manipulation technique will not be standardized, and the treating chiropractor can manipulate the lumbar spine if he/she determines to do so. The chiropractors record the types of treatment rendered at all sessions.

#### Advice group

Advice is given in an approximately 15-minute session following the baseline assessment, and is directed towards promoting self-management. The participants are told that their chest pain generally has a benign, self-limiting course. The participants receive individual instructions regarding posture and two or three exercises aiming to increase spinal or muscle stretch based on clinical evaluation. They are advised to seek medical attention for re-evaluation (family physician, chest pain clinic or emergency department) in case of severe or unfamiliar chest pain. Further, the advice group is asked to refrain from seeking any manual treatment for the following four weeks.

### Outcome measures

The outcomes are measures by self-report questionnaires that are collected at baseline, after four weeks (CTA positive patients only), and after three and 12 months (all patients) (see Figure 1).

#### Primary outcome measures in the RCT

Participants are asked to rate their worst level of chest pain during the last week, using an ordinal 11-point box scale (0 = no pain, 10 = the worst pain possible). Improvement in chest pain is rated by participants on a 7-point ordinal scale with responses ranging from "much worse" to "much better".

#### Secondary outcome measures

• Ordinal 11-point box scales (0 = no pain, 10 = the worst pain possible) will be used to rate chest pain "now" (i.e. on the day of examination/completion of questionnaire) together with the following types of pain over the last week: "worst" and "average" chest pain, "average" thoracic spine pain, "average" cervical spine pain, "average" shoulder and arm pain.

• Improvement in chest pain and general health is rated by the participants using an ordinal 7-point scale using the categories: "Much worse", "worse", "a little worse", "no change", "a little better", "better", and "much better".

• The general health status is measures by the Medical Outcomes Study Short Form 36-item Health Survey (SF-36) [31,32]. The SF-36 comprises 36 items that can be combined into eight multi-item summary scores: physical functioning, vitality, bodily pain, mental health, social functioning, role limitation due to physical health and due to emotional problems, and general health perception, plus one item assessing a change in health over the past year.

• The Patient Specific Functional Scale is developed to assess functional limitations in a variety of clinical presentations [33]. Participants will be asked to identify three important activities with which they are having difficulties or are unable to perform because of their problem. In addition to specifying the activities the participants will be asked to rate on an ordinal 11-point box scale the current level of difficulty associated with each activity.

• As a surrogate for the assessment of pain and quality of life, we will use the indicators "number of visits to family doctor", "number of hospitalizations", and "amount of prescribed drugs". The data will be obtained from the comprehensive national Danish central registers. Non-prescription medication use for chest pain is measured using self-report questionnaires at 12 and 52 weeks.

• Information about adverse events and side effects will be collected for the therapy group by the treating chiropractor before and after each treatment session.

### Predictors of outcomes

• Prior to commencing treatment, patients are asked to rate their expectation towards treatment benefits on a 5-point scale, with responses ranging from "getting much worse" to "getting much better".

• The Brief Illness Perception Questionnaire (B-IPQ) assesses perception of illness by asking patients for their own belief about their condition [34]. The B-IPQ consists of eight items that can be combined into five cognitive components: Identity, cause, time-line, consequences and cure/control. These components together make up the patient's perception of their illness. All eight items are measured using ordinal 11-point box scales.

### Health care cost/Cost-effectiveness analysis

Direct health care cost, direct non-health care cost and indirect cost are used in the economic evaluation as an indicator of cost-effectiveness. Cost data are collected through patient self-report questionnaires at 12 and 52 weeks. Direct costs for each patient will represent the one-year aggregated chest pain related health care costs based on utilization and estimated costs. Health care utilization (within and outside the study) is measured using standardized clinician treatment forms (each chiropractic visit, weeks 1–4), and patient rated self-report questionnaires (baseline and weeks 12 and 52). Direct health care costs include costs related to study treatment, non-study health care health provider use, medication utilization, and hospitalizations for chest pain. Indirect costs of productivity loss is measured by patient self-report (weeks 12 and 52) using questions that measure lost or impacted work or activity days due to chest pain. The EuroQol 5D (modified version) [35,36], a multi-attribute, patient self-report utility scale measuring five dimensions (mobility, self-care, usual activities, pain/discomfort, and anxiety/depression), is used as the cost-utility index. It is measured at baseline and weeks 4, 12, and 52.

### Data analyses

#### Diagnostic study

All items of the examination protocol will be compared between CTA positive and CTA negative patients. In order to show the importance of any single variable in the decision making process the variables will be compared both within all included patients and in subgroups. Using the Classification and Regression Trees (CART) method [37] the decision making process will subsequently be reconstructed into a decision tree for predicting continuous dependent variables (regression) and categorical predictor variables (classification). The decision tree will be compared to the reconstructed decision process from the Christensen study comprising chronic chest pain patients [26]. The agreement between the old and the new decision tree will be analyzed. Further, the proportion of CTA positive patients within the group of patients with normal MPS will be compared to proportion in the group of patients with abnormal MPS.

#### Randomized controlled trial

The size of the study sample was estimated using data from the study by Christensen et al. [27]. In this study, patients with suspected chronic stable angina pectoris were included. Improvement in chest pain over the last two weeks was assessed using an ordinal 5-point box scale. Using these results, a sample of 120 patients will provide 81% power to detect a shift in the distribution of the improvement in chest pain from 0%/5%/25%/45%/25% to 1%/10%/40%/40%/9%, which corresponds to the findings in the study by Christensen et al. The two studies are not similar in terms of patient characteristics (chronic versus acute chest pain), rating scales (5-point box scale versus 7-point box scale) or assessment period (two weeks versus one week). Nevertheless, a sample size of 120 patients was deemed sufficient.

The baseline scores of the patient demographics (e.g. age, gender, duration and history of complaints), primary and secondary outcomes will be used to compare the two intervention groups. Differences between baseline and follow up measurements will be calculated and compared. If necessary, adjustment for baseline variable will be made, using analysis of covariance (ANCOVA). A confirmatory, secondary analysis using the repeated measures, multivariate analysis of covariance (MANCOVA) will be used as an overall test for differences between groups. This will include both the primary and secondary patient-rated outcomes. The statistical analysis will be performed on the basis of the intention-to-treat principle, i.e. patients will be analysed in the treatment group to which they were randomly allocated. Finally, based on a prior definition of success, numbers needed to treat will be calculated. Outcomes of patient rated improvement will be dichotomized and success will be defined as patients rating "better" or "much better".

#### Cost-effectiveness analysis

A cost comparison of the therapy and advice group will be performed using data on direct and indirect costs. Cost differences between groups will be estimated using regression analysis where all chest pain-related costs in a year are regressed on treatment. A cost effectiveness analysis, using a mixed model linear regression analysis, will be conducted to compare the interventions, using patient-rated pain as the effective measure. Finally, a cost-utility analysis comparing the interventions will be performed using the EuroQol 5-D.

## Discussion

This study is the result of a unique research collaboration between researchers with backgrounds in chiropractic, cardiology, nuclear medicine and biostatistics, and to our knowledge this is the first randomized clinical trial investigating the effect of manual treatment on chest pain of musculoskeletal origin.

The design of this study has been a challenging process since no standardized and validated outcome measures for chest pain of musculoskeletal origin exists. The study by Christensen et al. [27] formed the basis for the present study. However, methods and results from the Christensen study are not directly applicable in this study, mainly because of differences in the two populations in terms of pain duration and other characteristics. Patients with chronic chest pain often have repeated pain episodes of a relatively mild character, sometimes described as "discomfort" [21]. They may experience pain that is brought on in familiar situations and at an expected work load. This is in contrast to patients with acute chest pain, who often experience a very dramatic and intense pain episode, some for the first time, and the pain evokes considerable anxiety and fear of cardiac conditions. In order to adapt to the differences in populations, a pilot study comprising 36 patients was conducted to determine the population size, inclusion and exclusion criteria, questionnaires, logistics and the primary outcome measures in the RCT. Following this, the semi-structured interview and pain rating scales were adjusted.

### Diagnostic part

An important part of the diagnostic procedure in this study is founded on manual examination of the muscles and joints. Palpation used as a diagnostic tool for spinal dysfunction has been subjected to criticism because of poor reproducibility and validity [38]. One of the major problems with the validation of palpation is that there is no golden standard to directly validate the findings. In the present study, this problem is addressed by using MPS as a *by proxy *measure to indirectly validate the CTA diagnosis. This is based on the hypothesis that in this population, patients who are CTA positive most likely will have fewer abnormal MPS than CTA negative patients. Data from the pilot study suggest that approximately 40% are CTA-positive, 15% have abnormal MPS, and 7% have both abnormal MPS and are CTA positive.

### Outcome measures

We have chosen global perceived effect as one out of two primary outcome measures even though critique has been posted on the reliability and validity of global rating scales [39]. Global rating scales are typically correlated with the patients' present status and are not an unbiased measure of change. However, global rating scales are regarded as clinically relevant and valid, and responsive to measure patients' perceived recovery. The global rating scale was also chosen, because during the pilot study we found that pain intensity levels were relatively low compared to for instance patients seeking care for low back pain. Patients initially reported very high levels of pain which then spontaneous decline in intensity within a very short period of time (hours to days), rendering pain a less than optimal primary outcome measure. Finally, we found that the Patient Specific Functional Scale [33] would not make a good primary outcome measure because many patients experience a first time episode of chest pain and, thus, do not feel limited in their daily activities.

### Interventions

Chiropractic therapy may be an effective treatment for patients with acute chest pain, but this has only been investigated in one non-randomized trial [27]. A pragmatic approach was chosen for the therapy group. The exact content of chiropractic therapy may not be clear, and the potential active "ingredient" can not be known even after this trial is completed. The advantage of the pragmatic strategy is that if this trial provides evidence in favor of chiropractic therapy, the results can easily be implemented, but future trials will be needed in order to identify the specific components. Also, our design is not well suited to correct for attention bias. Advice was chosen as intervention for the second group, because it is intended to mimic usual care and will act as a control treatment. Chiropractors are an integrate part of the Danish primary health care system with approximately 15% of Danes consulting a chiropractor each year [40]. Patients that previously have received chiropractic treatment very often have specific expectation about what chiropractic treatment consists of. This means that choosing a sham or placebo treatment was not feasible in Denmark due to lack of naïve patients, and because masking of patients to a sham or placebo treatment would not be possible.

In this study we have focused on two conditions that may cause episodes of chest pain, i.e. ischemic heart disease and CTA. Many other conditions may be present in these patients that could cause chest pain. Optimally, a thorough follow up, including evaluation of esophageal-gastro-intestinal conditions, would have been preferable to potentially diagnose some of the CTA-negative patients, but due to limitations in funding and time restraints such evaluation has not been possible.

In summary, this article presents the rationale and design of a multi-purpose study consisting of a prospective diagnostic study, and an RCT, with a cost-effectiveness study alongside the central trial. It is anticipated to be completed in 2008, at which time the results will be made available. The first part of this study may potentially demonstrate that a chiropractor is able to identify a subset of patients suffering from chest pain predominantly of musculoskeletal origin among patients dismissed from an acute chest pain clinic with no apparent cardiac condition. The long term goal is to establish whether manual palpation may be used as a part of the clinical examination to screen patients allowing for improvement in referral patterns. Furthermore knowledge about the benefits of manual treatment in patients with musculoskeletal chest pain will inform clinical decision and policy development in relation to clinical practice.

## Competing interests

The author(s) declare that they have no competing interests.

## Authors' contributions

MJS is the study manager and was involved in the design of the study, secured funding for the study, wrote the firsts draft of the manuscript and participated in subsequent revisions. HWC participated in the design of the study, in particular with respect to the design of the physical examination protocol, and commented on the manuscript. JH participated in the design of the study, in particular with respect to the design of questionnaires and outcome measures, and commented on the manuscript drafts. WV participated in the design, especially regarding data analysis, and commented on the manuscript. PFHC and TH both participated in the design of the study, in particular with respect to the cardiologic and nuclear medicine aspects of this study, and commented on the manuscript. All authors read and approved the final manuscript.

## Appendix 1

### Myocardial Perfusion Scintigraphy (MPS)

All patients undergo an electrocardiographically gated rest MPS according to the rest part of a two-day protocol without attenuation correction [41]. ^99m^Tc-sestamibi (10 MBq. kg^-1^, maximum 1100 MBq) is given 20 min. after sublingual administration of 0.5 mg nitro-glycerine followed 30–60 min. later by imaging using a dual-head gamma camera. A semi-automatic quantitative interpretation of perfusion and functional data is carried out using standard processing software (Auto QUANT^® ^5.0.0) [42]. Abnormal segmental perfusion scores is computed in a 20-segment model using a 5-point perfusion scoring scale (0 = normal, 1 = equivocal, 2 = moderate, 3 = severe reduction of radioactivity, and 4 = absence of detectable tracer uptake in a segment based on a normal databases set up for each sex). The summed rest score (SRS) is obtained by adding the scores of each segment in the 20-segment model [43]. A study is judged abnormal if the sum of stress scores is = 4 with at least one segment having a score = 2. In case of an abnormal MPS at rest, additional stress imaging is carried out at least two days later using adenosine infusion 0.140 mg. kg^-1. ^min^-1 ^for six minutes and injection of 10 MBq. kg^-1 ^(maximum 1100 MBq) of sestamibi between the third and the fourth minute of infusion. Similarly, from the images, the summed rest score (SRS) is calculated [43]. All studies are interpreted without knowledge of the clinical findings. In patients with both rest and stress images the type of abnormality is categorized as follows: Defects that are present at rest and remains unchanged during stress are considered as fixed. The appearance of new or worsening defects following stress is considered to be defect reversibility. Studies combining fixed and reversible defects are categorized as "reversible". The diagnostic accuracy of the MPS method has been reported elsewhere, and the estimated sensitivity and specificity for detecting significant coronary disease is 75% (95% CI 66%–82%) and 79% (95% CI 73%–84%), respectively [44].

## Pre-publication history

The pre-publication history for this paper can be accessed here:


